# A randomized controlled trial of tea tree oil (5%) body wash versus standard body wash to prevent colonization with methicillin-resistant Staphylococcus aureus (MRSA) in critically ill adults: research protocol

**DOI:** 10.1186/1471-2334-8-161

**Published:** 2008-11-28

**Authors:** Gillian Thompson, Bronagh Blackwood, Ronan McMullan, Fiona A Alderdice, T John Trinder, Gavin G Lavery, Danny F McAuley

**Affiliations:** 1 Regional Intensive Care Unit, the Royal Hospitals, Belfast Health & Social Care Trust, Belfast, Northern Ireland, UK; 2 Nursing & Midwifery Research Unit, School of Nursing & Midwifery, Queen's University Belfast, Belfast, Northern Ireland, UK; 3 Department of Microbiology, the Royal Hospitals, Belfast Health & Social Care Trust, Belfast, Northern Ireland, UK; 4 Intensive Care Unit, Ulster Hospital, South Eastern Health & Social Care Trust, Belfast, Northern Ireland, UK; 5 School of Medicine, Queen's University Belfast, Belfast, Northern Ireland, UK

## Abstract

**Background:**

Over the past ten years MRSA has become endemic in hospitals and is associated with increased healthcare costs. Critically ill patients are most at risk, in part because of the number of invasive therapies that they require in the intensive care unit (ICU). Washing with 5% tea tree oil (TTO) has been shown to be effective in removing MRSA on the skin. However, to date, no trials have evaluated the potential of TTO body wash to prevent MRSA colonization or infection. In addition, detecting MRSA by usual culture methods is slow. A faster method using a PCR assay has been developed in the laboratory, but requires evaluation in a large number of patients.

**Methods/Design:**

This study protocol describes the design of a multicentre, phase II/III prospective open-label randomized controlled clinical trial to evaluate whether a concentration of 5% TTO is effective in preventing MRSA colonization in comparison with a standard body wash (Johnsons Baby Softwash) in the ICU. In addition we will evaluate the cost-effectiveness of TTO body wash and assess the effectiveness of the PCR assay in detecting MRSA in critically ill patients. On admission to intensive care, swabs from the nose and groin will be taken to screen for MRSA as per current practice. Patients will be randomly assigned to be washed with the standard body wash or TTO body wash. On discharge from the unit, swabs will be taken again to identify whether there is a difference in MRSA colonization between the two groups.

**Discussion:**

If TTO body wash is found to be effective, widespread implementation of such a simple colonization prevention tool has the potential to impact on patient outcomes, healthcare resource use and patient confidence both nationally and internationally.

**Trial Registration:**

[ISRCTN65190967]

## Background

Over the past ten years there has been a dramatic increase in the number of infections caused by MRSA in several countries, including the United Kingdom (UK) [1]; this pathogen is now endemic in hospitals throughout the country. It appears that although many patients begin by being colonized, without any clinically evident infection, between 30% and 60% of colonized critically ill patients subsequently develop an infection syndrome attributable to the organism [2,3]. In fact, colonization pressure (number of MRSA carrier patient-days/total patient-days) is a major independent predictor of MRSA infection in a given critically ill population [4].

MRSA infection appears to be associated with greater attributable mortality than infection with methicillin-sensitive Staphylococcus aureus (MSSA), based on a recent meta-analysis of bacteraemia outcomes [5], although this may reflect the vulnerability of the patient population infected by MRSA, rather than greater virulence of the organism *per se*. Nevertheless, compared to MSSA, infections with MRSA demand treatment with antimicrobial agents of greater toxicity and acquisition costs; many of these drugs also require frequent monitoring of serum levels, further adding to the total cost of therapy [1,5]. Moreover, MRSA infection is associated with prolonged length of hospital stay, including duration of Intensive Care Unit (ICU) stay; using data from the National Audit Office, nosocomial infection is estimated to be associated with, on average, 11 additional days in hospital and total costs of 2.8 times that of uninfected patients [6-10]. Indeed, even without progression to infection, colonized patients require increased healthcare resource in the numerous measures implemented to limit the spread of MRSA to other patients. Investigators in Canada, using a cost model, estimated the additional cost per patient episode arising from MRSA colonization (without infection) equivalent to £650 [11]. Applying these costs to local ICU surveillance data, new MRSA colonization alone may add around £65,000 per annum to cost of care in a 17-bed ICU, aside from costs of prolonged ICU stay.

MRSA acquisition among critically ill patients represents a particular problem. Although it is difficult to compare data from different institutions and different countries because of inconsistencies in surveillance approaches, ICUs typically emerge with the highest incidence of MRSA compared to other wards [12]. Furthermore, ICUs may have a pivotal role in the dissemination of MRSA within an institution [13]. Moreover, prolongation of ICU stay attributed to MRSA infection has particular resource implications, arising from the exceptional cost of care in this setting [7]. Therefore, reducing the burden of MRSA in an ICU has potential to exert tangible benefits both within that ICU and in other clinical departments. Clearly, an intervention which might reduce the prevalence of MRSA has the potential to exert an extremely favourable impact on both patient outcomes and hospitalization costs.

Reducing the prevalence of MRSA among hospitalized patients requires several, complex, interrelated interventions. Fundamentally, such a strategy requires prompt identification of colonized individuals and robust methods to reduce transmission to noncolonized patients. In this study, we propose to address both of these requirements. First, in the form of a randomized clinical trial we aim to evaluate the impact of daily washing with a 5% tea tree (Melaleuca alternifolia) oil preparation on new MRSA acquisition among ICU patients. Second, in the form of a prospective observational study we aim to verify in this setting a rapid molecular assay for the detection of MRSA, with reference to extant culture-based methods.

Tea tree oil (TTO) is a naturally occurring chemical with broad microbicidal activity which is known to include MRSA [14,15]. Only a small number of clinical trials have been conducted using preparations containing TTO. These establish proof of concept that topical formulations suitable for human use may eradicate MRSA skin colonization [16-18]. In a randomized controlled trial (RCT) of TTO (4% nasal ointment and 5% body wash) compared to routine care (nasal mupirocin and triclosan body wash) for MRSA decolonization, 5 of 15 patients in the TTO group compared with 2 of 15 in the routine care group were successfully decolonized, although this difference was nonsignificant due to small sample size [17]. In the second published RCT for MRSA decolonization 224 patients were enrolled and received either standard treatment (nasal mupirocin and chlorhexidine body wash) or TTO (10% nasal cream and 5% body wash) [16]. Overall, 41% receiving TTO were successfully decolonized, similar to standard care. On subanalysis, TTO was superior for decolonization of superficial skin sites.

The utility of TTO preparations for MRSA decolonization has not been further evaluated in robust clinical trials. Furthermore, in spite of the laboratory and clinical evidence supporting the efficacy of such preparations in eradicating MRSA, there are no published data evaluating the role of TTO in the prevention of MRSA acquisition. Since TTO has proven efficacy, low toxicity and, as it is not part of the standard therapeutic armoury, prophylactic use does not expose a current MRSA treatment to the threat of resistance, we propose that this would be a logical strategy to investigate. In view of the resource implications of MRSA colonization and infection, it is imperative that interventions which focus on reducing these are also subjected to cost-effectiveness analyses.

With reference to identifying colonized patients more promptly, we propose to verify an established in-house MRSA PCR assay. This is an area of immense need since the current standard of culture-based diagnosis, with a turnaround time of 48–96 hours (depending on the result) is suboptimal. Therefore, noncolonized patients may be at risk of MRSA acquisition arising from exposure to colonized (but still unidentified) patients for an unacceptably long period of time. We have optimized a real-time PCR-based assay in our laboratory which has the potential to facilitate a same-day turnaround for MRSA detection. The assay, however, needs to be verified using a large number of clinical specimens from a large number of patients, with reference to the current diagnostic standard, before it is acceptable to introduce this new test to routine care.

## Methods

### Research hypotheses

The primary research hypothesis is that MRSA colonization among critically ill patients will be reduced by daily washing with 5% TTO body wash in comparison with a standard body wash (Johnsons Baby Softwash). The secondary hypotheses are that 5% TTO body wash is more cost-effective than a standard body wash (Johnsons Baby Softwash); and that there is no difference in sensitivity and specificity between a PCR-based assay for detection of colonization with MRSA and the standard culture method.

### Design and setting

This multicentre phase II/III prospective, open-label, randomized controlled clinical trial tests the effect of 5% TTO body wash versus standard body wash for prevention of MRSA colonization in critically ill adults. The study is being undertaken in two ICUs in two Health and Social Care Trusts (HSCT) in Northern Ireland. The Regional Intensive Care Unit (RICU) at the Belfast HSCT is a 17-bed intensive care unit (ICU) which receives general medical and surgical patients as well as patients both from regional specialty departments of the Royal Hospitals and from district general hospitals requiring level 3 care. The ICU at the South Eastern HSCT at the Ulster Hospital site is a 10-bed critical care unit which receives general medical and surgical patients requiring level 2 and level 3 care.

### Participants and sample size

All patients admitted to the ICUs during the study period will be eligible for inclusion in the study except those patients who fulfil the following exclusion criteria: less than 18 years of age; known to be colonized with MRSA at the time of admission; on admission, are judged as unlikely to remain in the ICU for at least 48 hours; are recruited, but whose pre-intervention MRSA screening tests are subsequently found to be positive (by standard culture); consent declined; and have a known sensitivity to TTO.

The primary outcome measure is the difference in MRSA colonization between groups at ICU discharge. The incidence of MRSA colonization during ICU admission in the study population is 13% (data obtained from the feasibility study conducted in one of the ICUs). A sample size of 1080 subjects (540 in each group) will have 80% power at a two-tailed significance level of 0.05 to detect a clinically significant difference of at least 40% in efficacy between 5% TTO and standard body wash. As the study will be confined to ICU, with nursing staff administering the study treatment, compliance will be guaranteed. Given the intensive monitoring in ICU, we do not expect any loss to follow-up.

### Recruitment of patients, randomisation and consent

When a patient is admitted, the investigator will contact the on-line randomization centre in the Clinical Research Support Centre [a centre providing design, management and coordination services for clinical trials and other studies conducted within the Health and Personal Social Services in Northern Ireland and funded by the Research and Development Office, Northern Ireland]. The investigator will complete the screening form on-line and this will generate a screening number indicating whether the patient is randomized or excluded. The randomization centre will provide the investigator with the allocation to receive either intervention A or B. The investigator will then obtain the appropriate intervention for the patient, which will be stored on the ICU. A record of all patients not randomized, including the reason for not being recruited, will be maintained by the investigator.

**Figure 1:**
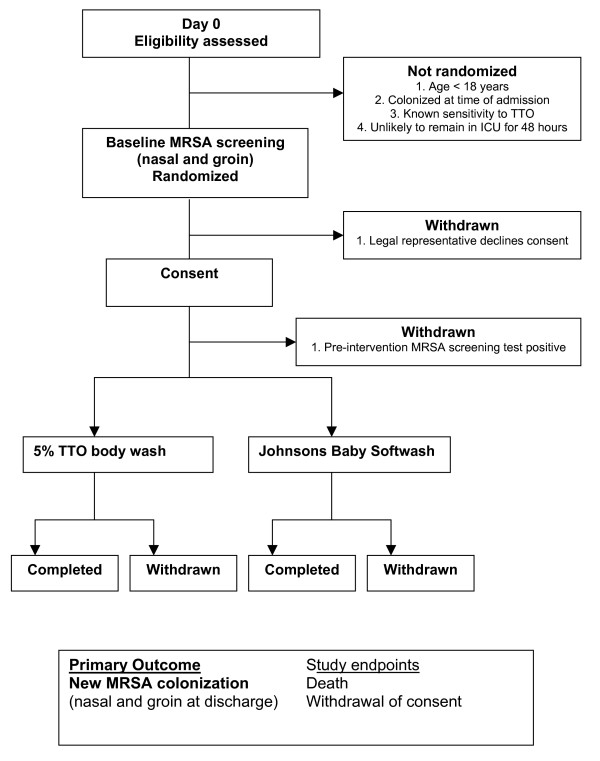
Participant enrolment flow diagram.

As patients are at risk from immediate exposure to MRSA from admission to the ICU, the success of this study will be dependent on randomizing patients as soon as possible. Furthermore as patients will be critically ill, they will not have the capacity to be able to give or withhold informed consent. Delay in recruiting patients would mean a significant proportion of patients would be excluded. Therefore, as the intervention is low risk, we plan to randomize patients on admission and obtain prospective/retrospective consent from their legal representative as soon as possible. In addition, retrospective consent will be obtained from the patient as soon as they are competent. This approach has previously been adopted in trials in the critical care setting [19-21]. In line with The Medicines for Human Use (Clinical Trials) Regulations 2004 and to comply with the Research Governance Framework, consenting processes will be standardised, and reinforced via training prior to study start-up. The planned flow of patients through the study is illustrated in Figure 1.

### Withdrawal from the study and the intervention

Individual patients will remain in the study until one of the following study termination criteria is reached: death; pre-intervention MRSA screening test is subsequently found to be positive; unit discharge; or patient or legal representative decline consent or request withdrawal from the study. The intervention will be continued in participating patients until one of the following intervention termination criteria is reached: adverse effect arising from either preparation; discharge from the unit; or MRSA colonization.

### Outcome measures

The primary outcome measure is new MRSA colonization during the inpatient episode in ICU, as defined by detection of MRSA by conventional culture methods in screening swabs of nose and groin, or in clinical specimens processed by the laboratory in the course of normal clinical care. This outcome measure is important as infection is typically preceded by colonization. The definition of colonization is clearly defined and unambiguous and it has established resource implications.

The secondary outcome measures are as follows: cost savings per case of MRSA colonization avoided; incidence of MRSA bacteraemia in each group; consumption of glycopeptide antibiotics, linezolid, rifampicin and fusidic acid; maximum increase in sequential organ failure assessment (SOFA) scores during inpatient episode, with reference to admission baseline; and agreement between MRSA screening results obtained by conventional culture and the PCR assay under evaluation.

### Ethical approval

The study will be conducted in accordance with the ethical principles that have their origin in the Declaration of Helsinki. It has been reviewed and approved by the Office for Research Ethics Committees Northern Ireland (reference 07/NIR03/71) and has received approval from the Medicines and Healthcare products Regulatory Agency (reference 22761/0008/001-0001).

### The Intervention

Intervention A is a standard care body wash preparation (Johnsons Baby Softwash). Intervention B is a proprietary 5% tea tree oil (TTO)-enriched body wash preparation, since this formulation has proven efficacy in achieving MRSA decolonization [16]. Patients will be randomised to 5% TTO body wash (Novabac 5% Skin Wash) or Johnsons Baby Softwash. The Novabac 5% Skin Wash contains the active ingredient melaleuca alternifolia oil 50 mg/g. When participants are allocated to either intervention the nurse will have only the allocated preparation available for the entire duration of time in the ICU that they are enrolled in the study. A standardized washing protocol is followed for patient hygiene in the ICUs. However, the quantity of body wash to be applied to individual patients is likely to be variable, depending on several factors that cannot be easily controlled, such as body surface area. The quantity administered therefore will be allocated to the discretion of the nursing staff.

### Risks to patients from intervention

The risk to patients associated with each intervention is estimated to be low. This is based on data assimilated in a recent review of the toxicity of TTO which concludes that topical use of this agent is relatively safe and that adverse effects are minor, self-limiting and uncommon [22]. Furthermore, in a randomized controlled trial of 110 patients who received 5% topical TTO for five days in a study of MRSA decolonization, no participants experienced adverse effects and no premature discontinuation was necessary [16]. Cutaneous adverse effects appear to occur in a significant proportion of patients only if much higher concentrations of TTO are used [23]. Very low concentrations (<0.01%) of TTO are associated with the development of resistance to both TTO and antibiotics in MRSA in vitro. However, as we will use 5% which kills MRSA this does not facilitate the development of resistance. Nonetheless, we will collect data on resistance of MRSA to antibiotics throughout the study. Adverse effects will be monitored daily by incorporating a record of washing and adverse events in standard nursing records. If any adverse events occur they will be reported to the medical staff and treated appropriately. An adverse events form will be completed. The Belfast, and South Eastern HSCTs are sponsoring and providing indemnity for this study. Participant safety and well-being are protected by implementing standard operating procedures as set out in the Research Governance Framework and the Medicines for Human Use (Clinical Trials) Regulations, 2004.

### Data collection methods and assessments: TTO evaluation and PCR verification

Data will be collected in a standardized manner across both sites according to Standard Operating Procedures. In both sites, patients will have screening specimens for MRSA carriage (using conventional culture methods on nasal and groin samples) carried out as a component of routine care by the nurse responsible for their care on admission to the ICU. For pragmatic reasons, duplicate specimens for processing by the PCR assay will only be taken in the RICU in the Belfast HSCT. All specimens will be obtained prior to either intervention commencing and will consist of swabs from both the nose and groin of all patients. Patients whose admission screening is positive by either method will be removed from the study (see exclusion criteria).

To facilitate verification of the PCR assay in the Belfast HSCT study population, its results will be compared to the culture results. Where there is disagreement between the culture and PCR results both will be repeated. In instances when the culture is persistently positive but the PCR assay persistently negative, this will be recorded as a false-negative PCR result (assuming a true-positive culture result). In instances when the culture result is persistently negative but the PCR assay is persistently positive, this will be recorded as a true-positive PCR result (assuming a false-negative culture result). If either test is not consistently positive or negative, it will be recorded as equivocal.

The presence of established major risk-factors for MRSA will be recorded, so that each can be analyzed as a covariate. These are:

• Total number of antibiotic-days during the ICU episode [24-26].

• Age (blocked by 10-year intervals).

• Number of comorbidities

• Intravascular catheter-days (number of devices × number of days) [27,28].

• Length of ICU stay [28,29].

• Severity of illness on admission to ICU [29].

Several other MRSA risk factors exist, such as transfer from high prevalence clinical units, multiple prior hospitalizations and residence in a nursing home. However, if participants are culture-negative at the time of admission to ICU, then these factors are not significant and therefore will not be recorded.

End-of-study screening specimens will be obtained from all participants on ICU discharge or death to identify those patients in whom MRSA acquisition has occurred during the inpatient episode. Additional information regarding MRSA acquisition as a result of MRSA detection in other clinical specimens taken as part of routine care in the ICU will also be recorded. The time of swab collection will be recorded.

Quantity of body wash will be determined by counting the number of bottles used. Antibiotic consumption will be retrieved from prospectively generated computerized patient records. Compliance and adverse effects will be monitored daily by incorporating a record of washing (including time of washes) and adverse events in standard nursing records.

### Cost-effectiveness evaluation

In the cost-effectiveness analysis, the evaluation will be performed from the perspective of the payer (i.e. the NHS), as advocated by the National Institute for Health and Clinical Excellence (NICE) [30], hence the evaluation will focus on additional direct healthcare costs incurred as a result of MRSA colonization. Collection of all resource use data will take place continuously throughout the study period. Patient-specific direct healthcare resource use (such as additional ICU days, antibiotic consumption, additional contact precautions, laboratory tests) will be collected alongside the RCT for both control and intervention groups, and supplemented with standardized unit cost data [31]. We will also record TISS (Therapeutic Intervention Scoring System) data. TISS is recognized and used worldwide as an indicator of the amount of care invested for a particular patient over an agreed time period. The score is a set of selected therapeutic activities among the many activities performed in ICUs. No attempt will be made to quantify non-healthcare costs, costs incurred by the patient, and costs associated with the rise of antibiotic resistance, as this is beyond the remit of the current evaluation.

### Schedule for assessments

Table 1 demonstrates the schedule for assessments to be performed at given time periods. Data for the previous 24-hours will be collected between 8 am and 10 am. The baseline assessment serves two purposes – first to ensure that patients recruited to the trial meet the inclusion criteria, and second to obtain baseline measures. Trial specific data will be collected in a Case Report Form for source data verification.

**Table 1:** Timing of assessments

	Baseline	Day 1 to *n*	Day *n *(ICU discharge)	Hospital discharge
Inclusion/exclusion criteria	*			
Demographics	*			
APACHE II score	*			
SOFA score	*	*	*	
TISS score		*	*	
Isolation	*	*	*	
Steroid use	*	*	*	
Antibiotic list	*	*	*	
Invasive devices list	*	*	*	
Colonisation data	*	*	*	
Infection data	*	*	*	
No. of cultures sent to laboratory	*	*	*	
Adverse events		*	*	
Whether neighbouring patient has MRSA		*	*	
Standard groin swab	*		*	
Standard nasal swab	*		*	
PCR groin swab (Belfast HSCT)	*			
PCR nasal swab (Belfast HSCT)	*			
Details of wash	*	*	*	
No of body wash bottles used		*	*	
Duration of ventilation			*	
ICU length of stay			*	
ICU survival			*	
Hospital length of stay				*
Hospital survival				*
Discharge location				*

APACHE II – Acute Physiological & Chronic Health Evaluation II; HSCT – Health and Social Care Trust; ICU – Intensive Care Unit; MRSA – Methicillin-resistant Staphylococcus aureus; PCR – Polymerase chain reaction; SOFA – Sequential Organ Failure Assessment; TISS – Therapeutic Intervention Scoring System;

### Statistical analyses

#### Hypothesis 1: TTO evaluation

For continuously distributed outcomes, differences between groups will be tested using independent samples t-tests, analysis of variance (ANOVA) and analysis of covariance (ANCOVA) with transformations of variables to normality if appropriate, or non-parametric equivalents. Chi-square tests (or Fisher's Exact tests) will be used for categorical variables. Major risk-factors for MRSA will be analyzed as covariates. Efficacy of intervention will be analysed on an intention to treat basis. A P value of ≤ 0.05 will be considered significant. A single final analysis is planned at the end of the trial.

#### Hypothesis 2: Cost-effectiveness analysis

Consistent with NICE recommendations, resource use and unit cost data will be presented separately, in £Sterling (2007); discounting of costs and outcomes at 3.5% per annum will be performed. Statistical analyses will be performed to examine differences in costs between groups and bootstrapping will be undertaken to allow for the skewed distribution of costs. Variability in cost-generating events will be examined by calculating the variance/mean ratio, and variability in costs examined by calculating the coefficient of variation. An incremental cost-effectiveness ratio (ICER) will be calculated, which is defined as the change in costs over the change in effect: ICER = Δc/Δe. The ICER (and 95% confidence ellipses) will be presented on the cost-effectiveness plane. Uncertainty relating to the data used in the analysis, methodological assumptions and the need to extrapolate the data or generalize to other settings will be addressed by decision analytic modelling and a range sensitivity analysis performed to determine the extent of uncertainty in point estimates.

#### Hypothesis 3: Verification of PCR assay

Agreement between MRSA screening results obtained by the PCR assay versus conventional culture with reference to the definitions of true and false positivity above will be evaluated, to estimate the sensitivity, specificity, positive and negative predictive values of the PCR assay in this population.

### Data Management and monitoring procedures

The study team will undertake the clinical trial in accordance with Good Clinical Practice. The data collected will be transcribed on to an electronic Case Report Form maintained by staff at the Clinical Research Support Centre. Site monitoring visits will involve source data verification. Submitted data will be anonymised, reviewed for completeness and consistency and then entered on a database. Data will be stored securely against unauthorised manipulation and accidental loss. Desktop security will be maintained through user names and frequently updated passwords and back up procedures. All essential documents and trial records will be archived in conformance with the applicable regulatory requirements and access to these archives will be restricted to authorised personnel.

### Safety and well being of study participants

Participant safety and well-being are protected by implementation of the sponsoring organisations' standard operating procedures as set out in the Research Governance Framework and The Medicines for Human Use (Clinical Trials) Regulations 2004. The research will be managed through a dedicated Research Management System. The conditional use of this system ensures compliance with sponsor and care/employing organisation responsibilities. The system utilises a risk assessment exercise within its study development phase and directs the focus and frequency of monitoring proportionate to study risk. Quality assurance procedures will be followed alongside mechanisms to ensure that all investigators can demonstrate that they are qualified by training and experience to fulfil their roles. Early termination of the study in response to safety issues will be addressed via the Data Monitoring Committee. Day to day management will be undertaken via a trials management group composed of the principal investigator and supporting staff. They will meet on a regular basis to discuss study issues. Site monitoring will be directed by the sponsor according to the study risk analysis.

### Safety of investigators

The University, the Belfast and the South Eastern HSCTs have Health and Safety Policies applicable to all employees. All personnel will adhere to any other Health and Safety regulations relating to their area of work. The principal investigator will ensure that all personnel have been trained appropriately to undertake their specific tasks. As the study fits closely to standard practice, additional risks to the investigators will be minimal. The study team will complete Good Clinical Practice and consent training prior to start up.

### Data Monitoring Committee (DMC)

A DMC will be convened by the Clinical Research Support Centre and will meet every six months. An interim analysis of efficacy is not planned although this can be requested by the DMC as required. The DMC will function primarily as a check for safety, reviewing adverse events. Early termination of study in response to safety issues will be addressed via the DMC. They will report any issues pertaining to safety to the Chief Investigator. It will be the responsibility of the principal investigator to inform the sponsor who will take appropriate action to halt the trial if concerns exist about participant safety.

## Discussion

The impact of MRSA on morbidity and mortality has been well documented, and TTO has been shown to eradicate MRSA skin colonization. However, intervention studies using preparations of TTO are not common. To our knowledge, the proposed research is the first study to evaluate the effectiveness of TTO body wash in preventing colonisation with MRSA in a critically ill patient cohort. This is particularly important because critically ill patients are more vulnerable to infections and ICUs have the highest incidence of MRSA colonization when compared with all other wards. The provision of such a therapy may reduce their chances of subsequently developing an MRSA infection.

The significance of this research can be considered from a number of aspects pertaining to the patient. With its focus on early identification and prevention, this study will determine the efficacy of a new method for early identification of MRSA, thus enabling prompt isolation to reduce transmission to non-colonized patients. It will also determine whether a simple body wash prevents patients from being colonised with MRSA. Finally, the research findings will be of value to clinicians and health service managers because they may facilitate more appropriate and efficient use of health care resources to improve outcomes for critically ill patients.

If the study findings are positive and conclusive, this intervention could be adopted widely – not only in ICUs, but also within the primary and secondary public and private health service institutions, residential and nursing homes. The innovative aspects of the research are in their application to this important group of vulnerable patients. This research will also inform clinicians and health service providers about an intervention and method that serves the needs of critically ill patients.

## Abbreviations

HSCT: Health and Social Care Trust; ICER: Incremental cost-effectiveness ratio; ICU: Intensive Care Unit; MRSA: Methicillin-resistant Staphylococcus aureus; MSSA: Methicillin-sensitive Staphylococcus aureus; NICE: National Institute for Clinical Excellence; PCR: Polymerase chain reaction; TTO: tea tree oil.

## Competing interests

The authors declare that they have no competing interests.

## Authors' contributions

GT conceived of the study and participated in its design; BB, FAA, RMcM and DFMcA have made substantial contributions to conception and design, obtaining funding and providing doctoral supervision; JT and GGL have made substantial contributions to design. All authors helped to draft the manuscript and have read and approved the final manuscript.

## Pre-publication history

The pre-publication history for this paper can be accessed here:


